# Patterns of Complementary Feeding Behaviors Predict Diet Quality in Early Childhood

**DOI:** 10.3390/nu12030810

**Published:** 2020-03-19

**Authors:** Karen M. Switkowski, Véronique Gingras, Sheryl L. Rifas-Shiman, Emily Oken

**Affiliations:** 1 Division of Chronic Disease Research Across the Lifecourse, Department of Population Medicine, Harvard Medical School and Harvard Pilgrim Health Care Institute, Boston, MA 02215, USA; Veronique_Gingras@harvardpilgrim.org (V.G.); sheryl_rifas@harvardpilgrim.org (S.L.R.-S.); emily_oken@harvardpilgrim.org (E.O.); 2 Department of Nutrition, Harvard TH Chan School of Public Health, Boston, MA 02115, USA

**Keywords:** complementary feeding, taste preference, diet quality, Youth Healthy Eating Index, latent class analysis

## Abstract

Infancy is a time of plasticity in development of taste preference. Complementary feeding (CF) may be a “sensitive period” for learning new taste preferences and establishing healthy dietary behaviors that may track later in life. Among 1162 children in the U.S. prospective cohort study Project Viva, we aimed to identify patterns of CF behaviors around 1 year and examine associations with diet quality in early childhood (median age 3.1y). We identified patterns of CF using latent class analysis (LCA) and examined later diet quality based on scores on the Youth Healthy Eating Index (YHEI). We identified four distinct CF patterns (latent classes). Later YHEI scores were highest in the class characterized by “breast milk and delayed sweets and fruit juice” and lowest in the “picky eaters” class. The classes defined as “late flavor introduction and delayed sweets” and “early flavor introduction and more fruit juice” had similar, moderate scores. Our results suggest that CF patterns that increase food acceptance and discourage the innate preference for sweetness may have persistent influences on diet quality.

## 1. Introduction

Diet quality is a major contributor to the risk of excess weight, a prevalent issue among U.S. children that may begin in infancy and track into adulthood [1]. By age two, food preferences and dietary patterns are established and may persist throughout childhood [2] and into adolescence and early adulthood [3]. Thus, there is a narrow window to establish preferences for healthy foods. The sense of taste develops as early as the third trimester of gestation, and babies are exposed to different tastes in utero (via amniotic fluid), through milk-feeding in infancy, and through complementary feeding (CF) [4,5], the introduction to different solid foods and liquids other than breast milk or formula [6]. Across cultures, babies show a distinct preference for sweet tastes and reject bitter and sour tastes, suggesting a biological basis for these preferences [7]. This preference for sweet taste results in natural acceptance of the sweet taste of breast milk and avoidance of toxins [5]. It also promotes higher consumption of energy-dense foods, which confers a survival advantage in food-scarce environment but is maladaptive for the modern food environment in developed countries such as the U.S. [8]. To establish a foundation for lifelong healthy eating habits, it is instead desirable to promote preferences for nutrient dense, whole foods over the refined, processed foods that young children naturally favor. 

Beyond the innate preference for sweet and energy-dense foods, infancy and toddlerhood is a time of plasticity in development of taste preference. The first few months of CF before a child has fully transitioned to the family diet may be a “sensitive period” for learning new preferences, as the brain and digestive system are still maturing and gut-brain connections are being formed [8,9]. Studies have suggested that the effects of foods and flavors encountered during CF may override any earlier preferences established through exposures to breast milk or formula. However, the window for introducing new tastes and textures is small, as children become neophobic at around two years of age and may be less receptive to trying new foods. Repeated exposure to a varied diet can increase acceptance and eventually liking of more nutritious foods such as fruits and vegetables [9]; conversely, repeated exposure to sweet foods in infancy and childhood compounds the natural preference for sweetness and creates an expectation of sweet taste [4]. 

Intervention studies conducted under carefully controlled conditions have demonstrated that early introduction of and repeated early exposure to a more varied, nutrient-dense diet increases acceptance of a variety of foods and can affect children’s future diet [10,11,12,13]. However, there is a lack of research in observational settings reflecting real-life conditions, in which different combinations of desirable and undesirable CF behaviors may occur. Additionally, much of the research has focused on very young babies or preschool-aged children, but little is known about the infant-toddler period, which may be the most critical window for shaping dietary preferences [8]. Previous research focused on the milk feeding period in infancy has demonstrated that this may be a sensitive period for the development of flavor preferences in both breastfed [14,15] and formula-fed [16,17,18] babies, but less is known about the impact of flavor exposure as a result of CF behaviors. There is also little research in U.S. populations, where CF feeding practices differ from other cultures. Finally, much of the research has focused on predictors of food acceptance, but it is not known if early influences have long-term associations with diet quality. 

The objective of this study was to identify CF behaviors, including timing of introduction of different food groups, early juice consumption, extended exposure to breast milk, and maternal perception of her child’s “pickiness”, that cluster in patterns (i.e. behaviors that tend to be observed together) using latent classes. We then aimed to examine whether the identified patterns (latent classes) predict later dietary quality, measured by Youth Healthy Eating Index (YHEI) scores. We hypothesized that late introduction of sweets and fruit juice and limited fruit juice intake at 1 year, early introduction of flavorful, non-sweet foods, extended breastfeeding, and continuing to offer foods initially refused by the child might co-occur and would be associated with a higher diet quality score, and that maternal perception of her child as a “picky” eater (disagreeing that her child likes fruits, vegetables, and new foods) would be associated with a lower diet score. Identification of behavior patterns associated with better child diet quality may help to inform future interventions targeting multiple CF practices.

## 2. Materials and Methods 

### 2.1. Subjects

We studied children enrolled in Project Viva, a prospective cohort study of mother-child pairs examining associations of prenatal, perinatal, and early-life exposures with pregnancy and child health outcomes. Project Viva recruited mothers in 1999–2002 at their initial obstetric appointment from eight offices of Atrius Health, a large multi-specialty group practice in eastern Massachusetts, U.S. Mothers enrolled their children after delivery. Project Viva collected data on the mothers via in-person study visits in the first and second trimesters of pregnancy and collected data on mothers and children at delivery and in infancy and early childhood at in-person visits. Data were also collected annually between in-person visits via questionnaire. We have previously described detailed recruitment and retention procedures [19]. 

Of the 2,128 Project Viva live singleton births, we included 1162 with relevant data from the 1-year questionnaire to identify patterns of CF behaviors (latent classes). Of these, 969 children had dietary data from the early childhood visit (median (IQR) age 3.1y (3.0–3.2y)) and were included for comparison of mean YHEI scores across latent classes. All mothers gave their informed consent for inclusion and that of their children before they participated in the study. The study was conducted in accordance with the Declaration of Helsinki, and the protocol was approved by the Institutional Review Board of Harvard Pilgrim Health Care (#235301). 

### 2.2. Exposure – Complementary Feeding Behaviors in Infancy

We identified patterns of CF using latent class analysis (LCA), a method that uses observed data from a set of indicator variables to group individuals into latent (unobserved) classes according to underlying patterns in their responses to the indicators [20]. We identified latent classes using responses to the 1-year questionnaire reported by the child’s mother. We included 11 indicator variables assessing the mother’s perception of her child’s food preferences, repeated offering of refused foods, timing of introduction of several complementary foods and food groups, and whether the child was primarily receiving breast milk vs. formula or some other type of milk during the CF period (9–11 months). 

### 2.3. Outcome – Youth Healthy Eating Index (YHEI) in Early Childhood 

We calculated the YHEI [21] total and component scores using dietary data collected via FFQ completed by the mother at the early childhood visit to report her child’s recent dietary intake. This FFQ was validated for use in preschool-age children [22] and assessed intake of different foods using questions in the format “Please check the box that best represents how often your child eats each of the foods listed, on average, in the past month.” For purposes of calculating the score, it was assumed that one “time” = 1 serving. We obtained information on frequency of fast-food consumption from a questionnaire administered at the early childhood visit. We had data available to calculate 10/13 components of the YHEI in early childhood. The three components that were not available are consumption of visible animal fat, eating breakfast, and eating dinner with family, all demonstrated to be minor contributors to variability in the total YHEI score in an external sample used for development of the score [21]. Additionally, we decided to omit the multivitamin component of the score since we were interested in examining quality of the child’s diet based on food intake. Thus, our total YHEI score included nine components with a range of possible total scores of 0–80 as detailed in Table S1.

### 2.4. Other Variables

We examined distributions of the following characteristics to describe our overall sample as well as the individual latent classes: child sex and race/ethnicity; maternal education level, pre-pregnancy body mass index (BMI) and diet score during pregnancy; household income at enrollment, and infant feeding method (partially or fully breastfed vs. formula-fed) at 6 months. Mothers reported their education level, pre-pregnancy weight, height and household income at the initial prenatal visit (median 9.9 weeks gestation). We calculated maternal pre-pregnancy BMI from these reports of height and weight. We obtained data on infant sex from hospital medical records, infant feeding from the 6-month interview, and child race/ethnicity from the early childhood interview. We measured maternal dietary quality during pregnancy using a slightly modified version of the Alternate Healthy Eating Index (AHEI) [23], calculated with comprehensive data collected from self-administered semi-quantitative food frequency questionnaires (FFQs) completed by mothers during the first and second study visits [24,25]. All data collection instruments used in Project Viva are publicly available at https://www.hms.harvard.edu/viva/. 

### 2.5. Statistical Analysis

To examine whether diet quality in early childhood was associated with patterns of CF in infancy, we compared latent class-specific mean total and component YHEI scores. First, we identified four latent classes of infant feeding patterns from the observed indicator variables. We recoded each variable to a binary variable indicating whether or not the CF practice was used, so that coefficients from the LCA model can be interpreted as odds of practicing the more desirable behavior. Table S2 provides a list of the 11 indicator variables, original response categories, and recoded response categories. We compared models with 2–5 classes and examined the G^2^ fit statistic from a likelihood ratio test, Akaike’s information criterion (AIC), Bayesian information criterion (BIC), and entropy R^2^ (an indicator of distinction between the latent classes) [26] for each model. We selected a four-class model of infant feeding patterns, which had good model fit, low AIC and BIC, high entropy indicating good distinction between the classes, and easy interpretability based on separation of indicators and clustering of related behaviors (Table S3). We named each class according to the distinctive behaviors of the class.

We used the modal assignment approach, which involves classifying each individual into his/her most likely class based on posterior probabilities, and assigned latent class was used as the exposure in our analysis. However, a simple comparison of means between assigned latent classes can be biased due to error inherent in assigning individuals to unobserved exposure classes, which may attenuate the observed estimates of association between exposure and outcome. Therefore, we used the “Bolck, Croon, and Hagenaars (BCH)” approach, which employs a weighted Analysis of variance (ANOVA) to obtain unbiased estimates using weights that are inversely related to the probabilities of classification error [27,28]. This has been shown to be a robust approach that produces unbiased estimates even when assumptions are violated [28]. We first examined results of the omnibus test comparing the expected YHEI scores for all four latent classes simultaneously, and if the null hypothesis of equality of mean scores across all latent classes was rejected (*p* < 0.05), we examined all pairwise comparisons between the four classes. In this study we were interested in predicting later diet quality from CF patterns identified through latent class analysis. Because latent classes are not an explicitly defined or directly assessed exposure, we were unable to identify clear potential confounders or attempt to answer a causal question. Therefore, we did not adjust our analyses for covariates. However, we did perform a series of sensitivity analyses comparing mean YHEI total scores across latent classes among strata of several variables (e.g. indicators of SES) that might reasonably be predictors of both latent class and diet quality to examine whether our results might be explained by associations of both the exposure and outcome with other factors. We performed calculation of the YHEI score using SAS version 9.4 (SAS Institute, Cary, NC, USA) and the latent class analysis and BCH weighting using Stata Release 15 (StataCorp, College Station, TX, USA) and the LCA Stata plugin [29] and LCA_Distal Stata function [30].

## 3. Results

### 3.1. Identification of Latent Classes of Infant Feeding Patterns

Overall and class-specific response probabilities for each of the 11 indicators are provided in Table 1. Overall, most of the mothers in the sample (≥90%) agreed that their child liked fruits and vegetables and that they continued to offer foods initially refused by the child, and 82% agreed that their child liked new foods. Less than half of the sample introduced eggs before 12 months, 36% introduced fish and only 16% introduced peanut butter. Over half of the sample (61%) reported that they introduced sweets at or after 12 months, but only 24% reported introducing fruit juice at or after 12 months and a similar proportion reported no fruit juice intake in the past month. At 9-11 months, 26% of the children were drinking primarily breast milk. 

We assigned a descriptive label to each class based on the marginal probabilities of an affirmative response to each indicator within each class. Class 1 (16% of the sample) was characterized by high probability that sweets and fruit juice were introduced at or after 12 months, high probability that the child did not drink any fruit juice in the past month, and relatively high probability that the child was receiving primarily breast milk at 9–11 months. We therefore designated this class as “breast milk and delayed sweets and fruit juice.” We defined Class 2 (7% of the sample) as “picky eaters” due to having low probabilities of the mother agreeing that her child liked vegetables and new foods and relatively low probability of agreeing that her child liked fruits. Class 3 (31% of the sample) was characterized by low probabilities of introducing fish, eggs, and peanut butter before 12 months and high probability of introducing sweets at or after 12 months; we labeled this class “late flavor introduction and delayed sweets.” Finally, Class 4 (45% of the sample) had the highest probabilities of introducing fish, eggs and peanut butter before 12 months, as well as the lowest probabilities that sweets and fruit juice were introduced after 12 months and that the child did not drink any fruit juice in the past month. We designated this class as “early introduction and more fruit juice.” 

Table 2 shows proportions of child and family characteristics in the overall sample and within each of the four latent classes. Class 1 had the highest proportion of children who: had college-educated mothers, came from households with an annual income >$70,000, were identified as being of white race/ethnicity, and were partially or fully breastfed at 6 months of age, and the lowest proportion of mothers with pre-pregnancy BMI in the obese category. Class 3 had the lowest proportion of children who were partially or fully breastfed at 6 months. Class 4 had the lowest proportion of children with college-educated mothers and who came from higher-income households. Approximately half of the sample was female, and the proportion of female children was similar among all latent classes. 

We had hypothesized that introduction of more foods, especially those with stronger flavors, during the CF period of approximately 12 months, as well as delayed introduction of sweets and fruit juice and longer exposure to breast milk, would be associated with better diet quality later in childhood. We also hypothesized that greater “pickiness” of the child perceived by the mother would be associated with lower diet quality. Therefore, after identifying the latent classes, we expected that membership in Class 1 would be associated with the highest total diet quality scores in early childhood, and that Class 2 would be associated with the lowest total diet quality scores. Classes 3 and 4 each exhibited a combination of more and less desirable behaviors, and therefore we expected moderate diet quality scores in these two groups.

### 3.2. Associations of Infant Feeding Patterns with Diet Quality in Early Childhood

The primary outcome was total YHEI score, a measure of overall diet quality in early childhood (median age 3.1 years, IQR 3.0–3.2y ). The overall mean total YHEI score in early childhood was 52.7 points (out of 80). Class-specific raw and BCH-adjusted total YHEI scores are presented in Table 3. Raw and BCH-adjusted total YHEI scores were very similar within each class and were highest in the “breast milk and delayed sweets and fruit juice” class (Class 1) and lowest in the “picky eaters” class (Class 2); the “late introduction and delayed sweets” and “early introduction and more fruit juice” classes (Classes 3 and 4, respectively) showed similar, moderate total scores. The BCH-adjusted comparison of mean YHEI scores indicated that YHEI scores in the “breast milk and delayed sweets and fruit juice” class were significantly higher than the scores for the other three classes (*p* < 0.01 for all, Table 3). Scores for the “picky eaters” class were also significantly lower than the scores for the “late introduction and delayed sweets” and “early introduction and more fruit juice” classes, and there was no difference between the scores in the latter two groups. To increase our confidence that the variability in diet quality was reflective of differences between the latent classes and not some other factor that was strongly predictive of CF patterns, we compared the BCH-adjusted YHEI scores between latent classes within strata of several different sociodemographic and behavioral variables, including maternal education level, BMI, and diet quality score during pregnancy and household income at enrollment. The results of these sensitivity analyses are presented in Table S4 and show that the same patterns of high mean YHEI score in class 1, low mean YHEI score in class 2, and moderate scores in classes 3 and 4 persist across different categories of participant characteristics.

**Figure 1:**
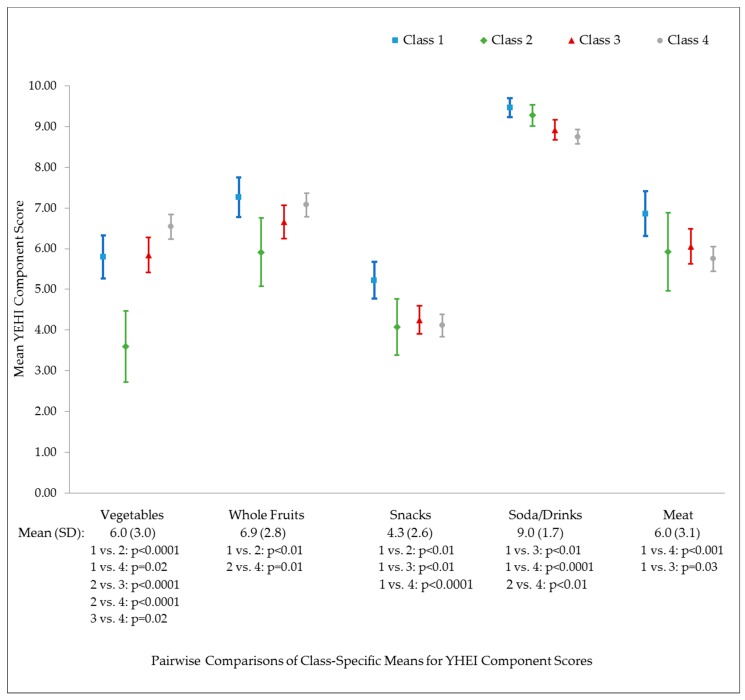
Comparison of adjusted ^1^ mean YHEI component scores across latent classes ^2^ among 969 Project Viva participants.

We also conducted a series of secondary analyses to determine whether different CF patterns in infancy were associated with specific dietary components in early childhood, measured using nine components of the YHEI as described above. Higher scores indicate better diet quality for all components. Results of the omnibus tests indicated no difference in mean score for the whole grains, dairy, margarine/butter and fast food components. Results for the other five components are shown in Figure 1. The highest mean score for the vegetable component was observed in the “early introduction and more fruit juice” class and the lowest score in the “picky eaters” class. The “picky eaters” class also had the lowest score on the whole fruit component. The “breast milk and delayed sweets and fruit juice” had the highest score on the snack component, with similar, lower scores observed in the other three classes. The highest scores on the soda/drinks component were observed in the “breast milk and delayed sweets and fruit juice” and “picky eaters” classes.

## 4. Discussion

Despite the importance of early exposure to different foods and tastes for shaping later preferences, there is a lack of established guidelines in the U.S. for what and how to feed infants and toddlers to promote healthful dietary habits [8]. Our results show that certain patterns of CF behaviors, as described by four latent classes in our sample, predict diet quality approximately 2 years later. We hypothesized that certain desirable behaviors would cluster together and predict higher diet quality in early childhood. Consistent with our hypothesis, we observed the highest mean total diet quality score among the latent class characterized as “breast milk and delayed sweets and fruit juice.” This class also had the highest scores on the snack, soda/drinks and meat ratio components of the YHEI, indicating that at around three years of age, they were consuming snack foods (including sweets) and non-nutritive drinks less frequently and consuming lean protein sources (in proportion to fatty protein sources) more frequently than children who were assigned to the other three latent classes. This could reflect a lesser preference for sweet-tasting foods and drinks in early childhood among this group, or it could simply be due to continued restriction of sweet foods and drinks by parents who did not introduce their child to these foods during the CF period.

However, we did observe that total YHEI scores were highest in this latent class among several subsets of participants, including children of mothers with both higher and lower diet quality (AHEI) scores during pregnancy and children who were still receiving breast milk at 6 months of age. This suggests that the observed association is not entirely due to associations with other factors reflecting the diet quality or nutrition/health knowledge of the family overall, which are expected to be associated both with CF practices as well as the child’s diet quality in early childhood when parents are still choosing which foods to offer to their child. Further research examining diet quality in later childhood and adolescence, when children have more autonomy over their diet and food choices, will help to further elucidate the relationship of CF practices with future diet quality. It is important to identify predictors of a dietary pattern characterized by lower consumption of sweets and non-nutritive drinks in early childhood, as we observed among this latent class. Nationally representative data from the U.S. Feeding Infants and Toddlers Study (FITS) and National Health and Nutrition Examination Survey indicate that preschool-aged children in the U.S. over-consume snack foods, desserts, and sweetened beverages, which is at odds with the need for a diet favoring nutrient-dense over energy-dense foods in this age group [31,32,33]. Our findings suggest that certain CF behaviors, including delaying the introduction of sweets and fruit juice, may be associated with less frequent consumption of these foods later on. This is in line with findings from the Australian NOURISH study, which found that wider exposure to “noncore” (nutrient-poor) foods at 14 months independently predicted greater preference for and intake of these foods in early childhood [34].

The lowest diet quality score was observed in the latent class characterized by mothers who perceived their children as picky eaters, based on low proportions of mothers who agreed that their child liked fruits, vegetables and new foods. The mean YHEI total score for this group was nearly 7 points lower than that the mean score for the group with delayed sweets and fruit juice. A recent narrative review examined differences in food and nutrient intakes between children perceived by their parents as picky eaters and those perceived as non-picky eaters and found that only intake of vegetables was lower among picky eaters [35]. Overall diet quality was not compared between the two groups in this review. Our results are consistent with the finding of more limited vegetable intake among picky eaters – the mean score for the YHEI vegetable component was significantly lower among the “picky eaters” class than the scores for each of the other three classes. In our study, infants in the “picky eaters” class also had lower intake of whole fruits in early childhood than infants in the other classes.

Interestingly, the “breast milk and delayed sweets and fruit juice” and “picky eaters” classes, which exhibited major differences in diet quality in early childhood, had similar proportions of mothers who reported that breast milk was the primary milk type provided to their infant during CF. This is in line with evidence suggesting that exposure to different flavors from the mother’s diet via breast milk may be important for initial acceptance of new foods at the beginning of CF, but that this effect is not sustained over time and other behaviors during the CF period are responsible for shaping later dietary preferences [4,9]. It is also possible that mothers who themselves eat a less varied diet (and consequently expose their children to less flavor variety during breastfeeding) are also more likely to characterize their infants as picky eaters and offer less food variety to the children as they get older.

Repeated exposure to sweet foods during CF can enhance children’s natural preference for sweet taste, leading to an expectation that food should taste sweet and rejection of foods with other taste profiles [4,5,36,37]. However, repeated exposure to a variety of flavors and textures, including fruits and vegetables, increases acceptance of new and previously rejected foods and flavors [4]. We did not observe a pattern characterized by the desirable combination of delaying introduction of sweets and fruit juice while introducing flavorful, non-sweet foods earlier. In our analysis, diet quality scores were very similar in Class 3, characterized by late (≥12 months) introduction of fish, eggs and peanut butter in addition to delayed introduction of sweets (but not fruit juice), and Class 4, which had the highest proportions of mothers who introduced fish, eggs and peanut butter before 12 months and the lowest proportion of mothers who delayed introduction of sweets. In other words, introducing more non-sweet flavor variety and also introducing sweets before 12 months resulted in a similar diet quality score as delaying introduction of the non-sweet, flavorful foods as well as sweets until at or after 12 months. This is an intriguing finding that we plan to explore in a future analysis in which we can examine the timing of introduction of sweets and of flavor variety as separate exposures. 

Strengths of our study include the wealth of detailed data on a variety of CF behaviors in over 1100 mother-child pairs, along with comprehensive dietary data on the majority of these participants two years later. We were able to identify four clearly interpretable latent classes of CF behaviors, with average posterior probabilities for class assignment of ≥82% in all 4 classes, indicating low probabilities of classification error (Table S5). In addition, we accounted for this potential classification error using the BCH method to obtain unbiased estimates of the differences in diet quality score across classes. We also examined differences in the individual components of the YHEI total score across latent classes to identify which specific components of the overall diet were associated with CF behaviors.

Our study also had several limitations. First, we had data available on the timing of introduction of various foods, but not on the frequency of offering these foods. Certain foods may have been introduced once and then not offered again, and our data do not capture that scenario. Additionally, we were unable to examine whether the timing of introduction of fruits, vegetables, and meat predicted later diet quality, as there was little variability in the timing of introduction of these foods within our sample. Many studies have demonstrated that repeated offering of foods initially refused by a child will eventually increase acceptance of the refused foods as well as other new foods. This should result in a healthier diet pattern, as foods such as fruits, vegetables, and whole grains will be more readily accepted, and children may be more receptive to a greater variety of foods. However, we were unable to examine this question in the present analysis as the proportion of parents who continued to offer initially refused foods was very similar across all latent classes. Our results suggest that certain patterns of CF behaviors are predictive of diet quality in early childhood, but this analysis was not designed to study a causal effect of these behaviors. It is possible that there are other factors, such as maternal education and nutrition knowledge, family socioeconomic status, and dietary practices and quality of the parents, which are associated with CF behaviors and also predict later diet quality and may explain the observed associations. We plan to examine these causal relationships carefully in a future study. However, in this study we conducted a sensitivity analysis in which we repeated the primary analysis in different strata of several potential confounding factors and observed similar results in the various subgroups. 

Additionally, we used questionnaires completed by the mother to assess exposure to different foods during CF and dietary intakes in early childhood, and thus our data rely on accurate reporting by the mother of her child’s food intake. The majority of our participants were of white race/ethnicity and came from households with annual income >$70,000 at enrollment and a college-education mother, which may limit the generalizability of our findings to other populations with more sociodemographic diversity. However, we did see very similar associations among various subsets of our sample, including children of nonwhite race/ethnicity, those from lower-income households, and those whose mothers did not have a college degree. Finally, each of the latent classes identified in our analysis included a mix of desirable and undesirable CF behaviors; there was no one class that was uniformly optimal in their CF practices according to our criteria, which were intended to capture exposure to different foods and flavors early in CF. Our future research on this topic will build on the observed associations of general patterns of CF behaviors with later diet quality and will attempt to identify effects of more specifically defined behaviors. Ultimately, we aim to identify early targets for intervention to promote healthful dietary patterns in childhood and adolescence.

## 5. Conclusions

In this study, we found that patterns of CF behaviors predict child diet quality approximately two years later. Membership in the class characterized by delayed introduction of sweets and fruit juice and low fruit juice intake at 1 year predicted higher diet quality at 3 years, while membership in the “picky eaters” class predicted lower diet quality. Feeding practices that increase food acceptance and discourage the preference for sweetness may have persistent influences on diet quality, but there is a need for further research on this topic.

## Figures and Tables

**Table 1:** Sample proportions in each class and overall and class-specific response probabilities ^1^ for latent class indicators measured at ~1y, among 1162 Project Viva participants.

Indicators	Overall	Class 1 (16%) “Breast Milk and Delayed Sweets and Fruit Juice”	Class 2 (7%) “Picky Eaters”	Class 3 (31%) “Late Flavor Introduction and Delayed Sweets”	Class 4 (45%) Early Introduction and More Fruit Juice”
Mother agrees that child likes fruits	96.8	96.4	70.1	99.4	99.5
Mother agrees that child likes vegetables	89.4	91.8	15.1	95.4	96.5
Mother agrees that child likes new foods	81.8	79.5	10.2	87.1	90.5
Mother continues to offer foods refused by child	92.5	92.0	91.5	91.4	93.6
Fish introduced before 12 m	36.2	40.0	17.1	7.8	57.5
Eggs introduced before 12 m	47.9	43.2	31.1	8.8	79.0
Peanut butter introduced before 12 m	15.6	4.8	16.7	2.5	28.1
Sweets not introduced before 12 m	60.9	78.4	55.3	84.2	39.7
Fruit juice not introduced before 12 m	23.6	87.2	17.6	16.1	7.2
Child did not drink fruit juice in past month	22.5	87.8	18.1	12.6	6.9
Child drank mostly breast milk from 9-11m	26.1	42.0	34.4	20.2	23.1

^1^ Light gray shading indicates class-specific probabilities >10% below the overall sample proportion, dark gray shading indicates class-specific probabilities >10% above the overall sample proportion.

**Table 2:** Demographic characteristics of 1162 ^1^ Project Viva participants – overall and by pattern of complementary feeding introduction (latent class).

	(%)				
Characteristics	Overall	Class 1 (16%) “Breast Milk and Delayed Sweets and Fruit Juice”	Class 2 (7%) “Picky Eaters”	Class 3 (31%) “Late Flavor Introduction and Delayed Sweets”	Class 4 (45%) “Early Introduction and More Fruit Juice”
Child sex					
Male	50.5	49.4	53.9	51.5	49.7
Female	49.5	50.6	46.2	48.5	50.3
Mother college graduate					
No	26.6	9.6	24.4	27.9	31.6
Yes	73.5	90.5	75.6	72.1	68.5
Household income at enrollment					
≤$70,000	32.4	20.1	35.2	28.2	39.0
>$70,000	67.7	79.9	64.8	71.9	61.0
Child race/ethnicity					
White	70.7	83.2	69.2	70.8	66.7
Non-white	29.4	16.9	30.8	29.2	33.3
Infant feeding at 6 m					
Formula only/weaned by 6 m	47.9	31.2	47.2	53.9	49.4
Partially/fully breastfed at 6 m	52.1	68.8	52.8	46.2	50.6
Mother pre-pregnancy BMI					
Underweight/normal weight	64.8	70.2	71.8	62.7	63.3
Overweight	22.3	21.9	15.4	24.9	21.7
Obese	13.0	7.9	12.8	12.4	15.0
Mother AHEI score in pregnancy					
Quartile 1	23.0	17.0	20.0	21.5	26.5
Quartile 2	24.7	24.3	28.0	22.6	25.7
Quartile 3	26.2	26.0	25.3	28.4	25.0
Quartile 4	26.1	32.8	26.7	27.5	22.8

^1^ Some of the 1162 participants were missing data on one or more of the variables included here.

**Table 3:** Comparison of raw and adjusted ^1^ mean total YHEI scores between latent classes among 969 Project Viva participants.

Overall Mean (SD) Total YHEI Score: 52.7 (9.3); Range: 20.4-76.5					
			Difference in Means (p-value)		
Class	Raw YHEI Score Mean (95% CI)	Adjusted ^1^ YHEI Score Mean (95% CI)	Class		
			2 ^3^	3 ^4^	4 ^5^
1 ^2^	55.2 (53.6, 56.7)	55.6 (53.8, 57.4)	6.62 (<0.0001)	3.40 (<0.01)	3.15 (<0.01)
2 ^3^	49.4 (47.1, 51.7)	49.0 (46.5, 51.5)		3.22 (0.03)	3.47 (0.01)
3 ^4^	52.4 (51.3, 53.4)	52.2 (51.0, 53.5)			0.25 (0.78)
4 ^5^	52.5 (51.7, 53.4)	52.5 (51.5, 53.5)			

^1^ Means were adjusted using the “Bolck, Croon, and Hagenaars (BCH)” approach, which applies weights that are inversely related to the probabilities of classification error to obtain unbiased estimates. ^2^ “Breast milk and delayed sweets and fruit juice” class. ^3^ “Picky eaters” class. ^4^ “Late flavor introduction and delayed sweets” class. ^5^ “Early introduction and more fruit juice” class.

## Supplementary Materials

The following are available online at https://www.mdpi.com/2072-6643/12/3/810/s1, Table S1: Calculation of the Youth Healthy Eating Index (YHEI) score in Project Viva, Table S2: Indicator Variables for Latent Class Analysis, Table S3: Comparison of models with 2–5 classes for 1162 Project Viva Participants, Table S4: Comparison of adjusted mean total YHEI scores between latent classes, stratified by sociodemographic and behavioral characteristics of Project Viva participants, Table S5: Average posterior probabilities for 4-class model
