# Supplementary material for: Patterns of Complementary Feeding Behaviors Predict Diet Quality in Early Childhood
Source: Nutrients. 2020 Mar 19;12(3):810. doi: 10.3390/nu12030810 (PMC7146403; doi:10.3390/nu12030810)
Supplement: Supplementary file 1 [file nutrients-12-00810-s001.pdf]

Table S1: Calculation of the Youth Healthy Eating Index (YHEI) score in Project Viva

#	YHEI Component	Max Score	Standard for Max Score	Min Score	Standard for Min Score	Included Variables
1	Whole grains	10	≥2	0	0	Brown rice, dark bread, hot cereal
2	Vegetables	10	≥3	0	0	Broccoli; cabbage, coleslaw, or cauliflower; carrots; corn; green beans; lettuce salad; potatoes (baked, boiled or mashed); mixed vegetables; peas (canned, frozen or fresh); peppers (green, red, or hot); spinach; squash (orange or winter); tomatoes; vegetable soup; sweet potatoes or yams
3	Fruit – whole fruits only, no juice	10	≥3	0	0	Apple or apple sauce; banana; strawberries or other berries; cantaloupe; orange or grapefruit; grapes; watermelon; peach or plum; pear; raisins or prunes
4	Dairy	10	≥3	0	0	Cheese; cottage cheese; cream cheese; ice cream; yogurt; milk, including chocolate milk
5	Meat ratio	10	≥2	0	0	Numerator: baked beans or chili beans; other dried beans, dried peas or lima beans; peanut butter; other (non-fried) chicken or turkey; canned tuna; other (non-fried) fish; tofu or soy beans; eggs; nuts<br>Denominator: beef – steak or roast; hamburger, meatballs, or meatloaf; fried chicken, chicken nuggets; fried fish, fish sticks; cold cuts (baloney, salami, ham); ham - baked or steak; hot dogs; pork - chops, roast, or ribs; sausage; bacon
6	Snack foods	10	0	0	≥3	Biscuit, cake or cupcake; chips (potato, corn or others); cookies or brownies; crackers, donut or fried dough; sweet roll or muffin; pie; chocolate candy; other candy; Jello; pudding
7	Soda and drinks	10	0	0	≥3	Fruit drinks (Hi-C, Kool-Aid, lemonade); hot chocolate; soda; sugar-free soda
8	Margarine and butter	5	Never	0	≥2 pats/day	Butter; stick margarine; tub margarine
9	Fast foods outside home	5	Never	0	Daily	N/A

Table S2: Indicator Variables for Latent Class Analysis

Question	Original Responses	Recoded Responses
1. My child usually likes fruits	1-Strongly agree<br>2-Agree<br>3-Disagree<br>4-Strongly disagree<br>5-Not applicable	1- Disagree/strongly disagree<br>2- Agree/strongly agree<br>Missing includes N/A
2. My child likes vegetables	1-Strongly agree<br>2-Agree<br>3-Disagree<br>4-Strongly disagree<br>5-Not applicable	1- Disagree/strongly disagree<br>2- Agree/strongly agree<br>Missing includes N/A
3. My child usually likes new foods	1-Strongly agree<br>2-Agree<br>3-Disagree<br>4-Strongly disagree<br>5-Not applicable	1- Disagree/strongly disagree<br>2- Agree/strongly agree<br>Missing includes N/A
4. If my child refuses to eat a new food, I continue to offer it to him/her at other times	1-Strongly agree<br>2-Agree<br>3-Disagree	1- Disagree/strongly disagree<br>2- Agree/strongly agree

	4-Strongly disagree<br>5-Not applicable	Missing includes N/A
5. How old was your baby when you first fed him/her…fish	1-Have not fed this to my child<br>2-Less than 6 months old<br>3-6 to 8 months old<br>4-9 to 11 months old<br>5-12 months or older	1- ≥12 months/not yet<br>2- <12 months
6. How old was your baby when you first fed him/her…eggs	1-Have not fed this to my child<br>2-Less than 6 months old<br>3-6 to 8 months old<br>4-9 to 11 months old<br>5-12 months or older	1- ≥12 months/not yet<br>2- <12 months
7. How old was your baby when you first fed him/her…peanut butter	1-Have not fed this to my child<br>2-Less than 6 months old<br>3-6 to 8 months old<br>4-9 to 11 months old<br>5-12 months or older	1- ≥12 months/not yet<br>2- <12 months
8. How old was your baby when you first fed him/her…sweets	1-Have not fed this to my child<br>2-Less than 6 months old<br>3-6 to 8 months old<br>4-9 to 11 months old<br>5-12 months or older	1- <12 months<br>2- ≥12 months/not yet
9. How old was your baby when you first fed him/her…fruit juice	1-Have not fed this to my child<br>2-Less than 6 months old<br>3-6 to 8 months old<br>4-9 to 11 months old<br>5-12 months or older	1- <12 months<br>2- ≥12 months/not yet
10. In the past month, how many ounces of fruit juice does your baby drink in an average day (24hour period)?	1-None<br>2-Less than 8 ounces<br>3-8 to 15 ounces<br>4-16 to 31 ounces<br>5-32 ounces or more	1- ≥0oz/day (some)<br>2- None
11. From age 9 through 11 months, which one of the following did your child mostly drink? (Check only one)	1-Formula [→D2a]<br>2-Breastmilk<br>3-Cow’s milk<br>4-Other milk (for example, goat’s milk)	1- Formula, cow’s milk or other milk<br>2- Breastmilk

Table S3: Comparison of latent class models with 2-5 classes for 1,162 Project Viva participants

Model	LL	G^2	AIC	BIC	aBIC	Entropy R^2
1 class	-6183	1509	1531	1587	1552	1
2 classes	-5987	1117	1163	1280	1206	0.55
3 classes	-5888	920	990	1167	1056	0.68
4 classes	-5769	681	775	1013	864	0.73
5 classes	-5750	642	760	1058	871	0.75

Table S4. Comparison of adjusted[1] mean total YHEI scores between latent classes, stratified by sociodemographic and behavioral characteristics of Project Viva participants.

Class	Adjusted[1] YHEI Score Mean (95% CI)	Difference in Means (p-value)		
		Class 2[3]	Class 3[4]	Class 4[5]
**Mother not a college graduate (N=237)**				
1[2]	54.8 (47.8, 61.8)	10.5 (0.02)	4.39 (0.27)	6.00 (0.11)
2[3]	44.3 (39.7, 49.0)		6.08 (0.03)	4.47 (0.09)
3[4]	50.4 (47.7, 53.1)			1.6 (0.38)
4[5]	48.8 (46.9, 50.7)			
Mother college graduate (N=731)				
1[2]	55.6 (53.8, 57.5)	5.4 (<0.01)	2.70 (0.03)	1.50 (0.18)
2[3]	50.2 (47.4, 53.0)		2.73 (0.10)	3.92 (0.01)
3[4]	52.9 (51.5, 54.3)			1.19 (0.23)
4[5]	54.1 (53.1, 55.2)			
Household income ≤$70,000 (N=295)				
1[2]	55.0 (52.9, 57.1)	5.33 (<0.01)	3.18 (0.02)	1.38 (0.28)
2[3]	49.6 (46.4, 52.9)		2.15 (0.25)	3.95 (0.03)
3[4]	51.8 (50.2, 53.4)			1.81 (0.11)
4[5]	53.6 (52.4, 54.8)			
Household income >$70,000 (N=628)				
1[2]	55.6 (53.8, 57.5)	5.4 (<0.01)	2.70 (0.03)	1.50 (0.18)
2[3]	50.2 (47.4, 53.0)		2.73 (0.10)	3.92 (0.01)
3[4]	52.9 (51.5, 54.3)			1.19 (0.23)
4[5]	54.1 (53.1, 55.2)			
Child white race/ethnicity (N=697)				
1[2]	55.9 (54.1, 57.7)	7.14 (<0.001)	3.29 (<0.01)	2.88 (<0.01)
2[3]	48.8 (45.7, 51.8)		3.86 (0.03)	4.26 (0.01)
3[4]	52.6 (51.1, 54.1)			0.40 (0.70)
4[5]	53.0 (51.9, 54.1)			
Child nonwhite race/ethnicity (N=272)				
1[2]	54.2 (48.4, 60.0)	4.34 (0.24)	2.79 (0.40)	2.79 (0.38)
2[3]	49.8 (45.7, 54.0)		1.55 (0.53)	1.55 (0.51)
3[4]	51.4 (49.0, 53.7)			0.00 (1.00)
4[5]	51.4 (49.5, 53.3)			
Infant fed formula only at 6m (N=437)				
1[2]	50.8 (47.6, 54.0)	4.82 (0.05)	0.74 (0.70)	0.29 (<0.01)
2[3]	46.0 (42.6, 49.4)		4.08 (0.04)	4.53 (0.02)
3[4]	50.0 (48.3, 51.8)			0.45 (0.72)
4[5]	50.5 (49.1, 51.9)			

Class	Adjusted[1] YHEI Score Mean (95% CI)	Difference in Means (p-value)		
		Class 2[3]	Class 3[4]	Class 4[5]
**Infant partially or fully breastfed at 6m (N=505)**				
1[2]	57.5 (55.4, 59.6)	5.67 (<0.01)	2.74 (0.06)	3.16 (0.01)
2[3]	51.9 (48.3, 55.4)		2.93 (0.15)	2.51 (0.73)
3[4]	54.8 (53.0, 56.5)			0.42 (0.73)
4[5]	54.4 (53.0, 55.7)			
Mother underweight/normal weight (N=635)				
1[2]	57.2 (55.3, 59.1)	7.40 (<0.001)	3.85 (<0.01)	4.62 (<0.001)
2[3]	49.8 (46.6, 52.9)		3.56 (0.05)	2.78 (0.11)
3[4]	53.3 (51.8, 54.9)			0.77 (0.49)
4[5]	52.6 (51.3, 53.8)			
Mother overweight (N=213)				
1[2]	51.7 (47.4, 55.9)	2.05 (0.51)	0.44 (0.87)	0.67 (0.79)
2[3]	49.6 (45.5, 53.7)		1.62 (0.52)	2.72 (0.25)
3[4]	51.2 (48.7, 53.8)			1.11 (0.55)
4[5]	52.3 (50.3, 54.4)			
Mother obese (N=118)				
1[2]	51.4 (42.9, 59.9)	7.68 (0.12)	2.70 (0.59)	0.80 (0.86)
2[3]	43.7 (39.4, 48.1)		4.98 (0.11)	8.48 (<0.01)
3[4]	48.7 (44.7, 52.8)			3.50 (0.18)
4[5]	52.2 (49.7, 54.7)			
Maternal diet quality (AHEI) score during pregnancy in Q1 or Q2 (N=454)				
1[2]	51.3 (48.5, 54.0)	5.84 (0.01)	1.26 (0.48)	1.52 (0.35)
2[3]	45.4 (42.0, 48.8)		4.57 (0.02)	4.32 (0.02)
3[4]	50.0 (48.1, 51.9)			0.25 (0.85)
4[5]	49.7 (48.4, 51.1)			
Maternal diet quality (AHEI) score during pregnancy in Q3 or Q4 (N=496)				
1[2]	58.7 (56.6, 60.8)	6.56 (<0.001)	4.68 (<0.01)	3.18 (0.01)
2[3]	52.2 (49.0, 55.3)		1.88 (0.31)	3.39 (0.05)
3[4]	54.1 (52.4, 55.7)			1.50 (0.20)
4[5]	55.6 (54.3, 56.8)			

Table S5: Average posterior probabilities for 4-class model

Class	Mean (range) _post_prob1	Mean (range) _post_prob2	Mean (range) _post_prob3	Mean (range) _post_prob4
1	.867 (.846-.889)	.021 (.011-.030)	.076 (.063-.089)	.036 (.027-.046)
2	.013 (.006-.020)	.904 (.884-.924)	.045 (.033-.057)	.038 (.019-.056)
3	.041 (.033-.048)	.019 (.015-.023)	.821 (.806-.837)	.119 (.106-.132)
4	.029 (.023-.036)	.008 (.005-.010)	.087 (.076-.098)	.876 (.863-.890)
